# Prognostic effect of HER2 evolution from primary breast cancer to breast cancer metastases

**DOI:** 10.1007/s00432-022-04486-0

**Published:** 2022-12-01

**Authors:** Sanja Löb, Eva Linsmeier, Saskia-Laureen Herbert, Tanja Schlaiß, Matthias Kiesel, Jörg Wischhusen, Jessica Salmen, Peter Kranke, Anne Quenzer, Florian Kurz, Claire Weiss, Elena Gerhard-Hartmann, Achim Wöckel, Joachim Diessner

**Affiliations:** 1 Department of Obstetrics and Gynaecology, University of Würzburg, Würzburg, Germany; 2 Department of Anaesthesia, Critical Care, Emergency and Pain Medicine, University of Würzburg, Würzburg, Germany; 3 Institute of Pathology, University of Würzburg, Würzburg, Germany; 4 Department of Obstetrics and Gynaecology, DRK Klinikum Westend, Berlin, Germany; 5 Universitäts-Frauenklinik Würzburg, Josef-Schneider-Straße, 4, 97080 Würzburg, Germany

**Keywords:** Breast cancer, HER2 conversion, HER2-low, Trastuzumab deruxtecan, HER2 targeted therapy, Trastuzumab

## Abstract

**Purpose:**

Therapeutic options for breast cancer (BC) treatment are constantly evolving. The Human Epidermal Growth Factor 2 (HER2)-low BC entity is a new subgroup, representing about 55% of all BC patients. New antibody–drug conjugates demonstrated promising results for this BC subgroup. Currently, there is limited information about the conversion of HER2 subtypes between primary tumor and recurrent disease.

**Methods:**

This retrospective study included women with BC at the University Medical Centre Wuerzburg from 1998 to 2021. Data were retrieved from patients' records. HER2 evolution from primary diagnosis to the first relapse and the development of secondary metastases was investigated.

**Results:**

In the HR-positive subgroup without HER2 overexpression, HER2-low expression in primary BC was 56.7 vs. 14.6% in the triple-negative subgroup (*p* < 0.000). In the cohort of the first relapse, HER2-low represented 64.1% of HR-positive vs. 48.2% of the triple-negative cohort (*p* = 0.03). In patients with secondary metastases, HER2-low was 75.6% vs. 50% in the triple negative subgroup (*p* = 0.10). The subgroup of HER2-positive breast cancer patients numerically increased in the course of disease; the HER2-negative overall cohort decreased. A loss of HER2 expression from primary BC to the first relapse correlated with a better OS (*p* = 0.018). No clinicopathological or therapeutic features could be identified as potential risk factors for HER2 conversion.

**Conclusion:**

HER2 expression is rising during the progression of BC disease. In view of upcoming therapeutical options, the re-analysis of newly developed metastasis will become increasingly important.

## Introduction

Breast cancer (BC) is the most common malignancy among women in Germany with about 70,000 new cases per year (Hubner et al. 2020). Four well-defined clinical subgroups, Luminal A, Luminal B, human epidermal growth factor receptor-2 (HER2) positive and triple negative (TN) show significantly different tumor growth and prognosis as well as therapeutic options (Perou et al. 2000; Szymiczek et al. 2021). HER2 is a member of the epidermal growth factor receptor family (Hwang et al. 2019). The overexpression of the HER2 receptor occurs in 15–30% of invasive BC and is associated with an adverse prognosis in early and advanced-stage breast cancer (Burstein 2005; Wolff et al. 2018). HER2-directed therapies in the neoadjuvant, adjuvant, and palliative setting have significantly improved the prognosis of patients affected by BC with HER2 amplification (Wynn and Tang 2022). With the advantage of effective anti-HER2 treatments in the last 2 decades, patients with HER2-positive tumors have the most favorable prognosis in early and metastatic situation (Hwang et al. 2019).

HER2 positivity is currently defined as 3 + HER2 expression in immunohistochemistry (IHC) or IHC 2 + and HER2 gene amplification measured by in situ hybridization (ISH) (Wolff et al. 2007a, b). Tumors with IHC 0, IHC 1 + , IHC 2 +, and ISH negative were clinically defined as HER2-negative, meaning that HER2 is neither amplified nor overexpressed. These patients were no candidates for anti-HER2 treatment with HER2-directed antibodies, antibody–drug conjugates (ADCs), or HER2 tyrosine kinase inhibitors (Wynn and Tang 2022). Nevertheless, BC with IHC 1 + and IHC 2 + demonstrate a significant level of HER2 expression on the cellular surface (Onsum et al. 2013). The assumption that this lower HER2 expression could also stimulate tumor growth encouraged researchers to treat these patients with anti-HER2 agents like trastuzumab and trastuzumab emtansine, but the results from different clinical trials were largely negative, indicating an insufficient therapeutic effect (Burris et al. 2011; Fehrenbacher et al. 2020). New ADCs such as trastuzumab deruxtecan and trastuzumab duocarmazine, however, demonstrated promising results for BC patients with HER2-low expression (Banerji et al. 2019; Modi et al. 2020). Trastuzumab deruxtecan is already approved by the US Food and Drug Administration (FDA) and the European Medicines Agency (EMA) for the treatment of HER2-positive metastatic BC that has already been treated with two or more lines of anti-HER2-based therapies (Gampenrieder et al. 2021). The DESTINY-Breast04 trial (ClinicalTrials.gov identifer: NCT03734029 and DB-06; NCT04494425), which focusses on HER2-low metastatic BC, could also present first positive data in comparison to treatment with chemotherapies of physicians’ choice. This advanced ADC has a homogeneous and high drug-to-antibody ratio of approximately eight molecules of cytotoxic agent per antibody. Using an elaborated cleavable linker, trastuzumab deruxtecan shows a so-called bystander killer effect and is assumed to target tumor cells with HER2 overexpression as well as BC cells with low HER2 expression (Beck et al. 2010; Gampenrieder et al. 2021). Based on this new therapeutic option, HER2-low BC entity should be classified as a new subgroup that may be amenable to these new anti-HER2 ADCs. The HER2-low subgroup, defined as IHC 1 + or IHC 2 + ISH −, comprises 50–55% of all BC patients and constitutes therefore a numerically large and significant group of patients (Gampenrieder et al. 2021; Schalper et al. 2014). This may be particularly relevant for metastatic BC, where new therapeutic options are most urgently needed. The discordance of hormone- and HER2-receptors of BC tumor cells between the primary tumor and different distant BC metastases is well described in the literature (Hoefnagel et al. 2013; Thompson et al. 2010). Yet, as there was no focus on the subgroup of HER2-low BC, there is only limited information about the conversion of the previously defined subtypes of the HER2 expression between primary tumor and recurrent disease. The aim of this analysis was therefore to investigate the possible switch between the different HER2 subgroups, with a focus on the HER2-low subgroup, and to evaluate possible influencing factors.

## Methods

### Study population

Stage IV breast cancer patients diagnosed between 1998 and 2021 at the University Medical Centre Wuerzburg were identified for the present study. Patients, who underwent tissue confirmation of the primary tumor and later received a biopsy of distant metastases with a complete HER2 status for both samples, were included in the study. Also, only patients with primary bc at the time of initial diagnosis were included in the present study. The follow-up data of these patients were collected from the national cancer register of the lower Franconian region Wuerzburg (Germany) including time of recurrence, overall survival, and last confirmed contact to the respective patient. These data were reviewed and completed according to the patients´ medical records, i.e., location of metastasis, method of biopsy, or surgical resection and pathological reports. Analysis of the overall population included menopausal status, age at diagnosis, histological grading of the tumor, BC subtypes, surgical interventions, type of adjuvant and palliative treatment, localisation of distant metastasis, time until relapse, and date of last contact or death.

### Pathology

HER2 status was tested according to published standards by ASCO/CAP using immunohistochemistry and fluorescence in situ hybridization (FISH) (Wolff et al. 2014, 2018). Tissue was collected in the course of surgery or biopsy, guided by sonography, computed tomography (CT), or magnetic resonance imaging (MRI). All procedures were performed as described in the Standard Operating Procedure (SOP) of the Institute of Pathology, University of Wuerzburg, Germany. Histological sections and immunohistochemical stainings were performed using formalin-fixed paraffin-embedded (FFPE) tissue slides according to the manufacturer’s instructions and standard protocols. The immunohistochemical staining was performed with the Ventana primary antibody, clone 4B5 according to the appropriate protocols within an automated immunostainer (Benchmark Ultra; Ventana/Roche, Tucson AZ, USA). If no membranous staining was observed, the sample was considered IHC 0/HER2-negative. Incomplete membranous staining that was faint/barely perceptible in > 10% of tumor cells was originally considered IHC 1 + /HER2-negative. The revised definition of IHC 2 + (equivocal) is invasive breast cancer with weak-to-moderate complete membrane staining observed in > 10% of tumor cells (Wolff et al. 2018). Tumors were classified IHC 3 + /HER2-positive if circumferential membrane staining was complete, intense, and within > 10% of tumor. All IHC 2 + tumors were further analyzed with FISH to determine the HER2 gene copy level using a HER2-specific probe and a centromeric probe [Kreatech™ FISH probes ERBB2 (17q12)/SE 17, Leica Biosystems, Germany] according to standard protocols and the manufacturer’s instructions. Tumors cells were evaluated and scored according to published standards by ASCO/CAP (Wolff et al. 2014, 2018); specifically, tumors were scored as HER2-positive if the HER2 gene-to-chromosome 17 ratio was ≥ 2.0 and cells had at a median value of ≥ 4.0 HER2 gene signals or if the HER2/CEP17 ratio was < 2.0, but the average HER2 copy number was ≥ 6.0.

For the present study, HER2-low tumors were defined as IHC 1 + and IHC 2 + in the absence of gene amplification examined by FISH. HER2-positive tumors were defined by IHC 3 + or IHC 1 + /IHC 2 + with a positive HER2 gene amplification in the FISH analysis. Finally, HER2-negative tumors were defined by IHC 0.

Hormone receptor (HR) status was considered positive in case of estrogen receptor (ER) and/or progesterone receptor (PgR) positivity, while HR status was classified as negative in case of negativity of both ER and PgR. Both ER and PgR were classified as positive in case of positive IHC staining in at least 1% of tumor nuclei (Allison et al. 2020; Wolff et al. 2014).

### Statistical analysis

Statistical analysis was carried out using IBM SPSS Statistics (version 27.0) software (IBM Corp, Armonk, NY, USA). Categorical data were described using numbers and percentages. Quantitative data were presented using median and range or mean and standard deviations. Statistical independence between variables was proved using *X*^2^ tests and for the frequency of less than 5 cells, Fishers’ test was utilized alternatively. McNemar chi-square test was applied to identify differences between connected values of repeated measurements, i.e., primary tumor and first as well as second metastasis. Overall survival (OS) was defined as the interval between detection of the primary tumor and death. Patients lost to follow-up were censored at the date of the last known contact. If no information was available, the status was coded as missing data. Survival distributions and median survival times were estimated using the Kaplan–Meier product-limit method. To determine possible influences on change of the HER2 receptor, logistic regression analysis was carried out after testing for multicollinearity. Univariate and multiple Cox proportional hazards regression was performed to determine possible influences on survival.

## Results

### Clinicopathological features of the patient cohort

**Fig. 1:**
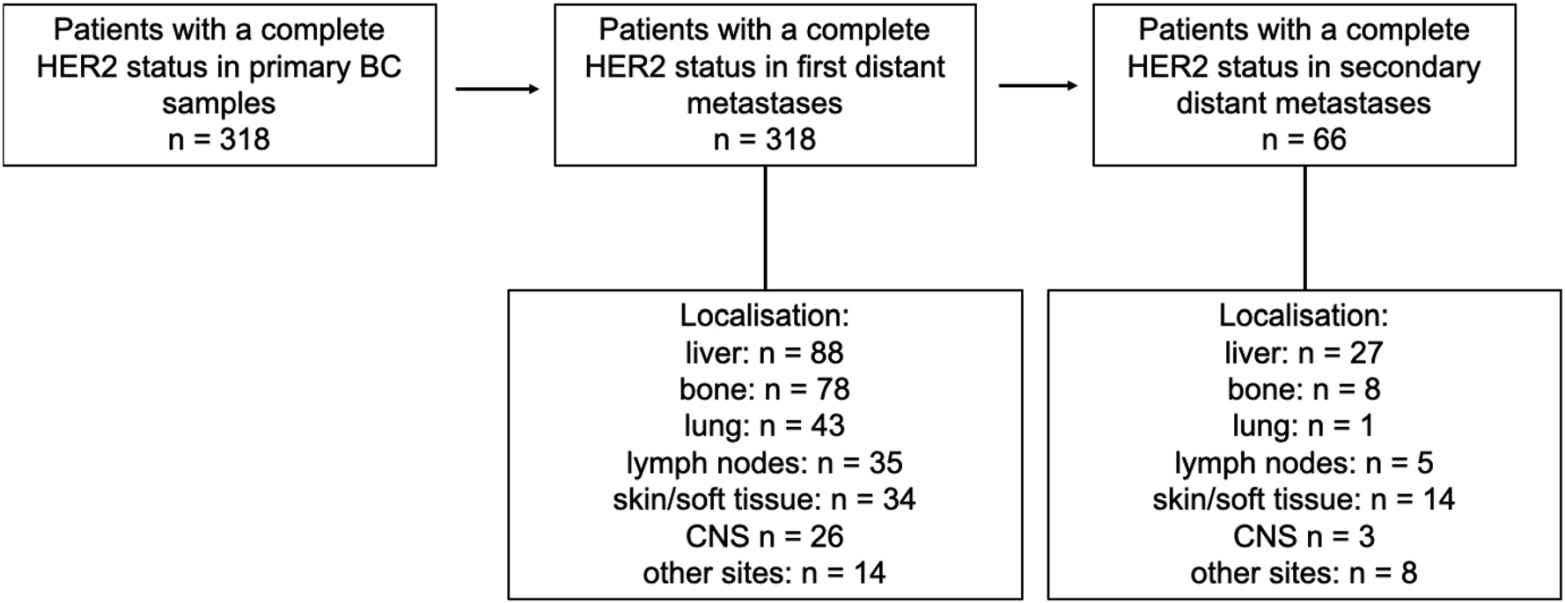
318 patients with a complete HER2 status on both primary and stage IV BC were included in the present study. 66 patients of the overall cohort, who developed metastases under palliative therapy, underwent biopsy of the new distant metastases. The localizations of the biopsies in metastatic BC are listed

A total of 756 patients with stage IV breast cancer were included in the present study. 318 patients underwent tissue confirmation of distant metastasis and presented with a complete HER2 status on both primary disease and the first relapse. 66 patients of the overall cohort, who developed metastases under palliative therapy, underwent biopsy of the new distant metastases. The time from diagnosis of primary BC and the first distant metastasis was 158.9 (mean) months and from primary BC to second distant metastases 202.9 (mean) months. The main clinicopathological features at diagnosis are summarized in Table 1. Figure 1 shows a flow diagram of the present study.

**Table 1:** Clinicopathological features of the study population

			Primary BC	First distant metastasis	Second distant metastasis
			*N* (%)		
Age at primary BC diagnosis (years, mean)	54.8 (26.6–87.0)				
Menopausal status	Premenopausal		129 (40.4)	73 (23.0)	14 (21.2)
	Postmenopausal		189 (59.6)	245 (77.0)	52 (78.7)
Histological grading	G1		15 (4.9)	6 (1.9)	2 (3)
	G2		159 (51.6)	48 (15.1)	3 (4.5)
	G3		134 (42.1)	55 (17.3)	11 (16.7)
	Missing		10 (15)	209 (65.7)	50 (75.8)
HR	Positive		261 (82.1)	240 (75.5)	47 (71.2)
	Negative		57 (17.9)	75 (23.6)	19 (28.8)
	Missing		0 (0.0)	2 (0.9)	0 (0.0)
HER2	HER2 0		126 (39.6)	98 (30.8)	14 (21.2)
	HER2 low		125 (39.3)	150 (47.2)	31 (47.0)
	HER2 +		67 (21.1)	70 (22.0)	21 (31.8)
Treatment	Chemotherapy	Yes	216 (67.9)	165 (51.9)	45 (68.2)
		No	101 (31.8)	153 (48.1)	20 (30.3)
		Missing	1 (0.3)	0 (0.0)	1 (0.5)
	Endocrine therapy	Yes	238 (74.8)	187 (58.8)	26 (39.4)
		No	80 (25.2)	131 (41.2)	39 (59.1)
		Missing	0	0	1 (0.5)
	Anti-HER2-therapy	Yes	53 (16.7)	70 (22.0)	19 (28.8)
		No	265 (83.3)	248 (78.0)	46 (69.7)
		Missing	0	0	1 (0.5)
Total			318 (100)	318 (100)	66 (100)

*BC* breast cancer, *HR* hormone receptor

The distribution of primary breast cancer subtypes was as follows: Hormone receptor (HR) positive without HER2 amplification or overexpression 66.1% (*n* = 210), HR-negative without HER2 amplification or overexpression (triple negative) 12.9% (*n* = 41), HER2-positive: 21% (*n* = 67). The phenotypes of the first metastasis distributed as follows: HR-positive without HER2 amplification or overexpression 60.3% (*n* = 192), triple negative 17.7% (*n* = 56), and HER2-positive 22% (*n* = 70). The distribution according to the tumor phenotypes of the second metastasis was: HR-positive without HER2 amplification or overexpression 50% (*n* = 33), triple negative 18.2% (*n* = 12), and HER-2 positive 31.8% (*n* = 21).

### HER2-low expression in primary and metastatic breast cancer

The proportion of the HER2-low subtype was 39.3% (*n* = 125) in primary BC samples, which accounts for 49.8% of the primary BC subgroup that was initially defined as HER2-negative. At the time of the first metastasis, 47.2% (*n* = 150) of the specimens expressed the HER2-low subtype. This accounts for 60.5% of the subgroup that had been formally classified as HER2-negative. Patients, who developed metastases under palliative therapy, expressed the HER2-low subtype in 47.0% (*n* = 31) of the cases. This represents 68.9% of the cohort that was deemed HER2-negative. HER2-low expression was investigated according to the breast cancer subtype (Table 2). We observed a continuous increase of HER2-low expression during the progress of BC. In the HR-positive BC subgroup without HER2 overexpression or amplification, we detected HER2-low expression in 56.7% of primary BC, in 64.1% upon first relapse and in 75.6% for the secondary metastases. In the triple negative subgroup, 14.6% showed HER2-low expression by the time of BC diagnosis. This proportion increased to 48.2% in specimens of the first metastasis and to 50.0% in samples of the second metastasis.

**Table 2:** HER2 distribution in the primary and metastatic breast cancer subgroups initially classified as HER2-negative (without HER2 overexpression or amplification)

	HER2-0 *n* (%)	HER2-low *n* (%)	*p*
**Primary BC**			
HR-positive without HER2 amplification or overexpression	91(43.3)	119 (56.7)	< 0.001
Triple-negative	35 (85.4)	6 (14.6)	
**First metastasis**			
HR-positive without HER2 amplification or overexpression	69 (35.9)	123 (64.1)	0.03
Triple-negative	29 (51.8)	27(48.2)	
**First metastasis with known second metastasis**			
HR-positive without HER2 amplification or overexpression	11 (31.4)	24 (68.6)	0.72
Triple-negative	2 (25)	6 (75)	
**Second metastasis**			
HR-positive without HER2 amplification or overexpression	8 (24.4)	25(75.6)	0.10
Triple-negative	6 (50.0)	6 (50.0)	

There is a continuous increase of HER2-low expression during the progress of BC. In patients without HER2 amplification or overexpression, HER-2 low expression is significantly more frequent (*p* < 0.001) in patients with primary HR-positive BC than in the triple negative cohort. In samples of first metastases from patients without HER2 amplification or overexpression, HER2-low expression also presented significantly elevated (*p* = 0.033) in HR-positive BC without HER2 amplification or overexpression when compared to the triple negative cohort. In patients with first metastases with known second metastases, there was no significant difference in HER2-low expression in the different BC subtypes. In patients with secondary metastases, there was also no significant difference in HER2-low expression in the different BC subtypes. HER2 0 = IHC 0; HER2 low = IHC 1 + or IHC 2 + with the absence of HER2 gene amplification by FISH

In patients without HER2 amplification or overexpression, HER-2 low expression is significantly more frequent (*p* < 0.001) in patients with primary HR-positive BC than in the triple negative cohort. In samples of first metastases, HER2-low expression also presented significantly elevated (*p* = 0.033) in HR-positive BC (without HER2 amplification or overexpression) when compared to the triple negative cohort. In patients with secondary metastases, there was no significant difference (*p* = 0.10) in HER2-low expression between the different BC subtypes.

### Factors correlating with HER2 evolution from primary to metastatic breast cancer

The evolution of HER2-receptor expression was analyzed from primary BC to metastatic BC and from the first metastases to the development to secondary metastases under palliative therapy.

In the subgroup of primary BC to metastatic BC, we identified an overall rate of HER2 conversion of 33.6% (*n* = 107). In 58.9% (*n* = 63) of the converted cases, there was a switch from HER2-0 or HER2-positive to HER2-low expression. In 14.8% of the cases, a change from HER2-0 to HER2-low expression was found, which accounts for 37.3% of the HER2-0 primary BC subgroup. Among the primary HER2-low BC cohort, 6.0% of the patients showed a loss of HER2 expression. In the primary BC HER2-positive subgroup (*n* = 67), a loss (*n* = 3) or downregulation (*n* = 16) of HER2 expression was observed in 19 patients, while 48 maintained an HER2-positive subtype.

**Fig. 2:**
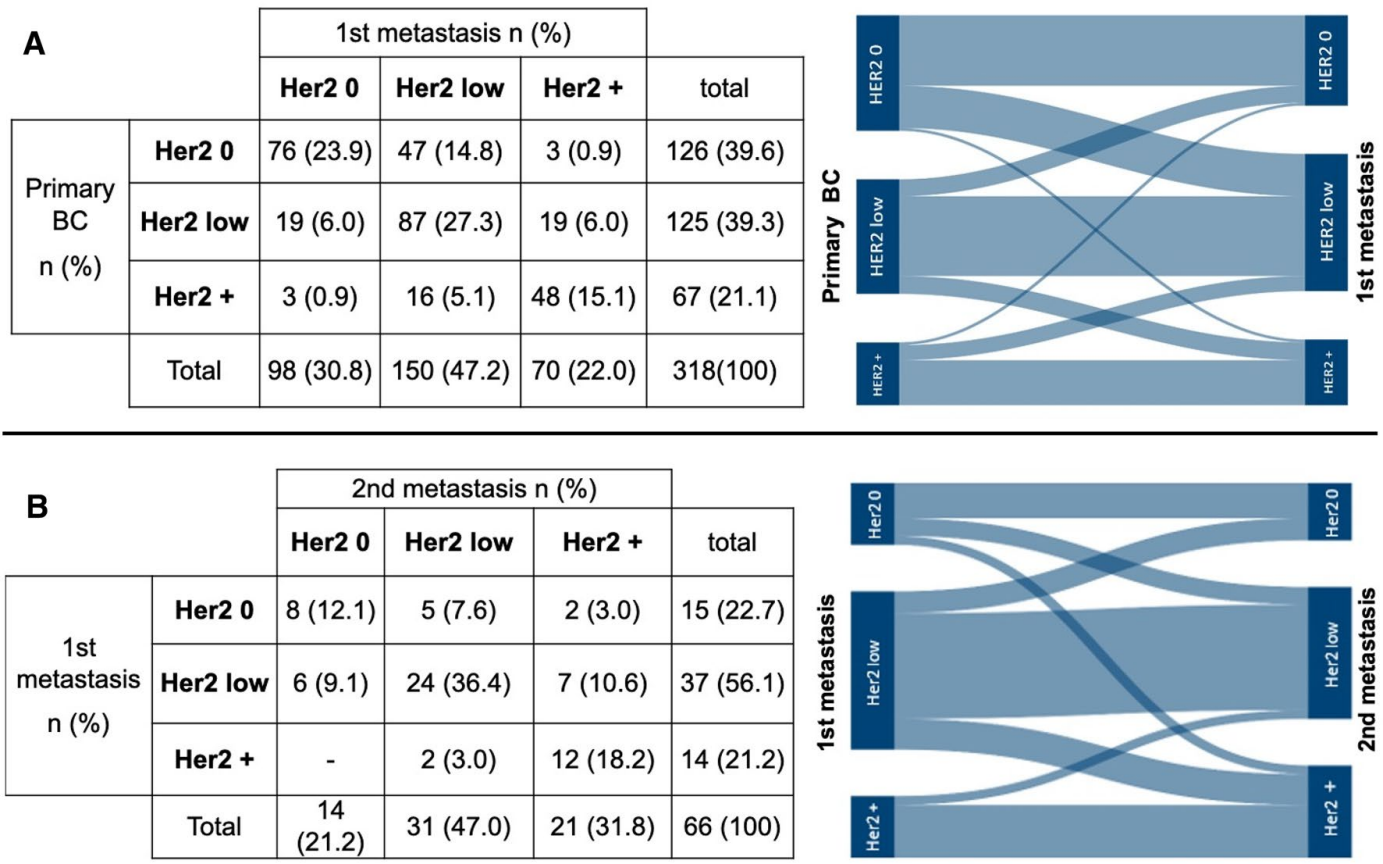
HER2 evolution from primary to metastatic breast cancer (BC). **A** Conversion of HER2 expression from primary BC to the first (1st) metastasis. **B** HER2 evolution from fist metastasis to secondary (2nd) metastases under palliative therapy. HER2 0 = IHC 0; HER2 +  = IHC 3 + or IHC 1 + /IHC 2 + with a positive HER2 gene amplification by FISH; HER2 low = IHC 1 + or IHC 2 + with absence of HER2 gene amplification by FISH

In the subgroup of first metastases to secondary metastases, there was an overall rate of HER2 evolution in 33.3% (*n* = 22) of the cases. In 31.8% (*n* = 7) of the converted cases, an evolution from HER2-0 or HER2-positive to HER2-low was observed. 40.9% (*n* = 9) became HER2-positive. In 9.1% of the patients with an HER2-low subtype in the first metastasis, there was a loss of HER2 expression to HER-0 in the second metastasis. The HER2-positive phenotype in the first metastasis maintained some level of HER2 expression in the second metastasis. The majority of the group remained HER2-positive (*n* = 12) (85.7% of the initial HER2-positive first metastasis cohort). Despite treatments with HER2-targeting agents, only two patients converted to an HER2-low phenotype (14.3% of the primary HER2-positive first metastasis cohort). The HER2 evolution from primary BC to first and secondary metastases is illustrated in Fig. 2.

Several clinical, pathological, and therapeutic features of the overall cohort were analyzed as potential factors influencing HER2-receptor conversion in the progress of BC. Age at primary BC diagnosis, menopausal status at primary BC diagnosis, histological grading of BC, different subtypes of BC (HR + /HER2-negative, triple negative, HER2-positive), and type of adjuvant and palliative treatments (endocrine therapy, endocrine therapy and chemotherapy, chemotherapy, anti-HER2-therapy). An association between one of these factors and HER2 evolution from primary BC to the first relapse and from the first relapse to secondary metastasis could not be identified (data not shown).

### Impact of HER2-evolution on overall survival (OS)

**Fig. 3:**
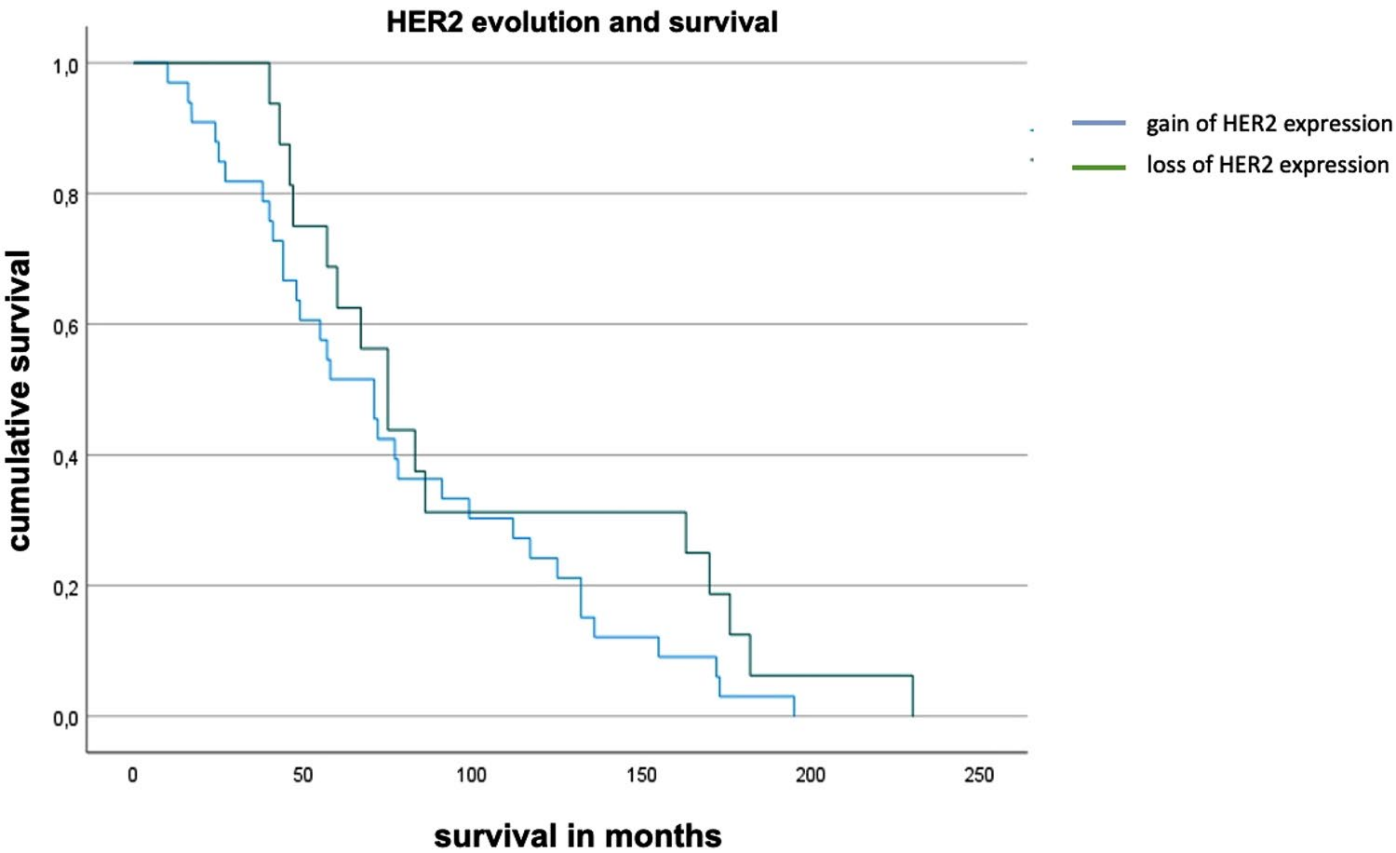
Kaplan–Meier analysis for OS regarding HER2 evolution from primary BC to the first relapse. The mean time of survival of patients with loss of HER2 expression (99.4 months (mean), 95% CI 69.1–129.7) differed from patients with gain of HER2 expression (77.8 months (mean), 95% CI 1856–2911) by 21.0 months. The difference was not significant (*p* = 0.177)

For correlation and survival analysis from primary BC to the first relapse, we defined two subgroups of HER2-receptor conversion. Patients, whose tumor increased the HER2 expression (*n* = 69) (HER2-0 to HER2-low or HER2-positive, HER2-low to HER2-positive), were included in the first subgroup. The second subgroup included patients with a loss of HER2 expression (*n* = 38) in the BC tumor (HER2-positive to HER2-low or HER2-0, HER2-low to HER2-0). Cox regression analysis showed that a loss of HER2 expression from primary BC to the first relapse correlated significantly with a better OS (hazard ratio 0.533, 95% CI 0.32–0.90, *p* = 0.018). Patients with loss of HER2 expression survived 99.4 months on average, whereas patients with a gain of HER2 expression survived 77.8 months after the diagnosis of metastatic BC (95% confidence interval of the mean: loss of HER2 69.1–129.7 months vs. gain of HER2 61.0–95.7 months). The survival times were compared by Kaplan–Meier analysis (Fig. 3). Patients with loss of HER2 survived 21.0 months longer in comparison to patients with a gain of HER2, but the difference was not significant (*p* = 0.177).

## Discussion

Human epidermal growth factor receptor-2 (HER2/neu or c-erbB2) is a proto-oncogene that mediates functions, such as proliferation, differentiation, and survival in malignant and normal breast epithelial cells (Gschwind et al. 2004). About 15–25% of all invasive breast cancers overexpress the HER2 protein, which is associated with an aggressive tumor phenotype and unfavorable prognosis (Cobleigh et al. 1999; Slamon et al. 1987). The humanized monoclonal antibodies trastuzumab and pertuzumab (Herceptin, Genentech, San Francisco, CA) as well as the antibody–drug conjugate T-DM1 (Kadcyla, Genentech, San Francisco, CA) have improved disease-free and overall survival of breast cancer patients in the adjuvant and metastasized setting (Cobleigh et al. 1999; Verma et al. 2012). In the past, the dichotomous classification of breast cancer into HER2-positive or HER2-negative defined the treatment and therapeutic algorithm of patients affected by breast cancer. Only patients classified as HER2-positive could benefit from this targeted therapy. HER2 positivity was defined as overexpression or amplification of HER2. Attempts to extend anti-HER2 therapy with trastuzumab to patients classified as HER2-negative were unsuccessful (Cobleigh et al. 1999; Fehrenbacher et al. 2020). The further development of the HER2-based antibody–drug conjugate trastuzumab deruxtecan is characterized by an enzyme-cleavable antibody–drug linker, high drug-to-antibody ratio, and membrane-permeable payload. Due to these properties, trastuzumab deruxtecan showed antitumor activity in breast cancer patients with low HER2 expression—formally characterized as HER2-negative (Modi et al. 2022). Based on the positive clinical results of the DESTINY-Breast04 trial, a precise differentiation of HER2 expression will become increasingly important, in particular for patients with breast cancer in an advanced stage (Lee and Park 2022; Modi et al. 2022). In the present retrospective, monocentric study, we evaluated the evolution of HER2 expression from the primary tumor in the breast to recurrent, metastatic breast cancer. Furthermore, we re-evaluated HER2 expression in newly developed metastasis during ongoing first-line metastatic therapy. In our overall study population, we analyzed the evolution of HER2 antigen expression at three different time points in the course of disease. Initially, we included 756 patients with metastatic breast cancer from our documentation system. 435 breast cancer patients had to be excluded afterward for this analysis. Reasons were in some cases a refusal to have distant metastases biopsied. In other cases, palliative therapy started based on the clinical diagnosis of stage IV breast cancer, or sufficient data were missing. For 318 patients, clinicopathological data with a complete, differentiated HER2 status based on IHC and ISH for the primary breast cancer and the recurrent disease or the first metastasis were available and could be included in this statistical evaluation. For another 66 patients, we could extract complete histological and clinical information for the second metastasis after primary palliative therapy.

We observed an increasing expression of the HER2 antigen during the course of disease of our breast cancer patients. This statistically significant trend could be reaffirmed for each subgroup analysis. The population of HER2-low patients with IHC 1 + and IHC 2 + with negative results on ISH grew from the primary tumor to recurrent disease and from recurrent disease to secondary metastasis in the entire study population (HER2-low: 39.3–47.0% − 47.2%).

For Luminal A and Luminal B breast tumors, which do not overexpress HER2, we detected an increase in the group of HER2-low breast cancers from 56.7 for the primary tumor to 64.1% for recurrent disease and to 75.6% for the secondary metastasis. The truly HER2-negative (HER2-0) group decreased accordingly. The subgroups of HER2-positive breast cancer patients numerically increase in the course of disease (HER2-positive: 21.1–22.0% − 31.8%), whereas the HER2-negative cohort decreases correspondingly.

This trend of increasing HER2 expression was also detected among triple negative tumors: the HER2-low subgroup increased from 14.6 for the primary tumor to 48.2% for recurrent disease and to 50% for the secondary metastasis.

Our results are in line with the literature. Miglietta et al. as well as Van Poznak et al. observed a higher proportion of HER2-low tumors among distant metastasis and local recurrence of breast cancers (Miglietta et al. 2021; Van Poznak et al. 2015). Moreover, in accordance with several publications, we could ascertain a higher prevalence of HER2-low tumors among hormone receptor positive/HER2-negative breast tumors in comparison to tumors with a triple negative phenotype (Miglietta et al. 2021; Rossi et al. 2012; Sapino et al. 2014; Schettini et al. 2021). In addition to the existing literature, we re-analyzed HER2 expression after the first line of metastatic therapy. Obviously, the number of patients decreased with each analysis. We could, however, demonstrate that the HER2-positive and HER2-low subgroup expanded, while the proportion of the HER2-negative population numerically decreased. In view of the limited number of patients in terms of the second analysis, it is crucial to verify this observation in further studies.

Focusing on the clinical, prognostic effect of HER2-low expression, several recent, retrospective studies showed conflicting data. Just as the proportion of HER2-low breast cancers varied from 16.2 (Jacot et al. 2021) to 64.4% (Horisawa et al. 2022), some studies showed improved survival for HER2-low patients whereas others described rather negative clinical effects (Alves et al. 2022; de Moura Leite et al. 2021; Horisawa et al. 2022; Tan et al. 2022; Won et al. 2022). In our analysis, we therefore focused on the evolution of HER2 expression from primary tumor to recurrent disease. A loss of HER2 expression in recurrent BC, e.g., HER2-positive to HER2-low or HER2-0, HER2-low to HER2-0, correlated significantly with a better OS (Hazard ratio 0.533, 95% CI 0.32–0.90, *p* = 0.018). Moreover, patients with a loss of HER2 from primary BC to recurrent disease survived 21.0 months longer in comparison to patients with a gain of HER2, but the Kaplan–Meier analysis showed that the difference was not significant (*p* = 0.177). We might hypothesize that there was no significant difference due to the size of the two subgroups, and therefore, studies with a larger validation cohort are needed.

As the HER2 oncogene has immunosuppressive characteristics in the tumor microenvironment and stimulates cell proliferation and differentiation, it is not surprising that an increasing expression is accompanied with a negative prognostic effect (Kirchner et al. 2021; Press et al. 1990). Based on the positive clinical results of the DESTINY-Breast04 trial, the detection of the variation of HER2 expression from early to advanced metastatic breast cancer will become increasingly important (Modi et al. 2022).

Being able to better define the population of patients that might benefit from targeted HER2 therapy with the antibody drug conjugate trastuzumab deruxtecan would facilitate the identification of the subgroup of breast cancer patients for whom a re-analysis of HER2 expression is particularly important. For a better understanding and assessment of the evolution of HER2 antigen expression during the course of disease, we therefore tried to detect clinical factors influencing HER2 expression. In multivariate regression analysis, we assessed the impact of clinical and therapeutically parameters, such as menopausal status, age, grading, hormone receptor status, proliferation markers, chemotherapy, targeted therapy (also with anti-HER2 agents), and antihormonal therapy on the shift of HER2 expression from primary tumor to recurrent disease. However, we could not detect a specific parameter or a combination of parameters influencing HER2 expression in a specific direction. These results underline the importance of re-testing antigen expression and, in particular, HER2 expression on tumor cells after tumor progression under ongoing therapy for currently all patients, in particular in view of new therapeutic options.

Focusing on the molecular breast cancer subtypes, we obtained conflicting results (Perou et al. 2000). For the primary tumor, we observed an association between Luminal B-like subtype and HER2-low expression, whereas for recurrent disease and second metastasis, HER2-low is dominant in the Luminal A-like subtype. For the HER2-negative subgroup, the results point in the opposite direction. Interestingly, Miglietta et al. also describe an association between Luminal B-like subtype and HER2-low expression among breast cancer patients, while Schettini et al. found a possible correlation between HER2-low tumors and Luminal A subtype in a retrospective study (Miglietta et al. 2021; Schettini et al. 2021). It has to be mentioned however, that in our study, as well as in the publication of Miglietta et al., the definition of the molecular subtypes is based on an IHC assessment of the Ki67 proliferation marker. Schettini et al. determined the molecular intrinsic subtypes using the PAM50-muligene signature instead (Schettini et al. 2021). The apparent lack of comparability may therefore be due to the variable definition of the intrinsic subtypes.

Investigating a potential correlation of the HER2-gene expression and the site of breast cancer recurrence or the second metastasis, we could not detect any significant associations (data not shown). These findings are in line with the results achieved by Miglietta et al. (2021). In view of the upcoming launch of the antibody–drug conjugate trastuzumab deruxtecan for HER2-low breast cancer patients, the missing correlation between different anatomic sites and the level of HER2 expression underlines the importance of a biopsy-based re-evaluation of HER2 expression of a breast cancer metastasis irrespective of the anatomical site. A limitation of our study might be the high dropout rate in the subgroup of patients who developed metastases under palliative therapy. This is largely explained by patients not consenting to another biopsy or a second line palliative therapy, and by patients dying before reaching the next line of therapy. However, the results regarding the evolution of HER2 expression in this subgroup are in line with the HER2 evolution from primary breast cancer to the development of first metastases. While these results certainly require confirmation in further studies with a larger study group, they already provide significant support for taking re-biopsies from patients under palliative therapy, who might then become eligible to treatment with trastuzumab deruxtecan.

One strength of the present work is the monocentric character of this study. Although we did not perform a central revision for HER2 expression for this study, all analyses were carried out by the same institute of pathology according to the current ASCO recommendations (Wolff et al. 2013; Wolff et al. 2007a, b). Furthermore, we analyzed HER2 and hormone receptor expression at the primary tumor, the first appearance of metastasis or recurrence and the second metastasis after primary palliative therapy. We can therefore describe HER2 antigen expression during the course of therapy taking clinical and therapeutically factors into account.

A limitation of this study worth noting is the lack of a formal definition of the HER2–low subgroup. Therefore, the identification of this subgroup is highly depending on the applied immunohistochemical and in situ hybridization protocols. For the present analysis, we used IHC and ISH to describe HER2-low breast tumors. According to this standardized protocol, HER2-low tumors were defined as IHC 1 + and IHC 2 + in the absence of HER2 gene amplification by ISH, following previous studies (Banerji et al. 2019; Denkert et al. 2021; Modi et al. 2022; Tarantino et al. 2022).

However, it has to be mentioned that the reliability of HER2 IHC and ISH is influenced by an inter-observer variability, formalin-fixation variables as well as intratumoral heterogeneity. Especially, the differentiation of IHC 0 and IHC 1 + can be inconsistent (Lambein et al. 2013; Marchio et al. 2021). Another strength of this analysis is the follow-up of 20 years that could be extracted from one database. At the same time, the long period of data analysis can also be seen as a limitation, since systemic therapies as well as therapeutically algorithms have changed significantly over the last 20 years. Another limitation of this analysis, that should be highlighted, is the divergence of the examined tissue. For the primary tumor, we used breast tissue obtained by a biopsy. For the recurrent disease and the secondary metastasis, we analyzed tissue most easily accessible to biopsy. We can therefore not exclude that HER2 expression might vary in another localisation of metastasis. It would be of interest to compare HER2 expression levels in different tissues with metastasis of breast cancer in the same line of therapy. In general, the fastest growing metastasis that can be biopsied was histologically confirmed and analyzed for antigen expression. Therefore, comparing tumor antigen expression at different localisations at the same time would require a prospective analysis.

## Conclusion

Our retrospective analysis has not only emphasized the dynamic and mutating character of breast cancer. Most importantly, our data show increasing HER2 expression during the course of disease of breast cancer from primary tumor to distant disease. In view of upcoming therapeutic options, this indicates that re-analysis of newly developed metastasis will become increasingly important for all breast cancer patients.
